# Telemedicine as an auxiliary tool in trichiasis treatment follow-up

**DOI:** 10.5935/0004-2749.2022-0137

**Published:** 2025-08-21

**Authors:** Guilherme Kamano, Filipe Lemos Bellote, Philipe Renee Rodrigues de Oliveira, Silvana Artioli Schellini

**Affiliations:** 1 Faculdade de Medicina, Universidade Estadual Paulista “Júlio de Mesquita Filho”, Botucatu, SP, Brazil

Dear Editor,

Trichiasis is a disorder of the eyelid margin characterized by misdirected eyelash growth toward the ocular surface, where they touch and irritate the cornea^(1)^. It is most commonly observed in older adults and the majority of cases are caused by persistent inflammation of the eyelid margin^(2)^. Laser thermal ablation is a rapid and easy treatment for milder manifestations of this condition and is recommended when there are 10 or fewer affected lashes. Following the treatment, a three-month follow-up is required to assess new lash growth^(3,4)^.

Our oculoplastic outpatient clinic generally assists patients living far from public healthcare facilities. This necessitates the expenditure of time and money when a patient simply requires a quick check to confirm their trichiasis has abated. To address this, we reasoned that since telemedicine has already been proven efficacious in various medical situations, most notably during the COVID-19 epidemic, it may also be used to track trichiasis patients^(5)^.

To assess the validity of this approach, we conducted a study with 39 trichiasis patients who had been treated with thermal laser ablation. We utilized parameters established in a previous study^(4)^. Our institution’s ethics committee approved the study protocol (Number: 16652919.0.0000.5411) and all participants provided written informed consent. Three months after laser treatment, a slit-lamp examination (10× magnification) was performed on each patient by one of the researchers and the results were compared to two sets of photographic images; one taken by the author and the other by a companion of the patient. The images were standardized and taken from each eye of each patient using smartphone cameras in primary gaze and supraversion position (upgaze), with free adjustment of luminance, distance, and focus to obtain the eyelid image best suited to trichiasis identification.

The images obtained were placed in random order and sent to two expert ophthalmologists for independent evaluations. The ophthalmologists conducting these assessments were instructed to look for the presence of trichiasis, and provide subjective ratings of the image quality based on the ease with which they were able to detect the presence or absence of trichiasis. Images were given a score between 0 and 5, with 0-3 denoting poorer image quality and 4-5, excellent quality. Statistical analyses of the responses of both ophthalmologists were then conducted using Cohen’s kappa to establish the level of interrater agreement, and the slit-lamp examination findings were used to determine the sensitivity of their evaluations^(6)^.

Assessments of both expert ophthalmologists exhibited low sensitivity to the accurate detection of trichiasis for all of the images taken (Table 1). Although the evaluator’s responses showed greater accuracy with McNemar’s test and Yates’s continuity correction (p<0.05), the kappa test revealed poor interrater agreement, both for the photos taken by the researcher (kappa = 0.3561) and those taken by the patient’s companion (kappa = 0.2110).

**Table 1:** Sensitivity values of evaluator trichiasis detection by gaze position, photographer, and assigned grade range

Variable	Evaluator 1 (s)	Evaluator 2 (s)
Supraversion position	30.00%	34.28%
Primary gaze position	22.85%	32.85%
Researcher photograph	40.00%	51.42%
Patient’s companion photograph	12.85%	15.71%
Grade range 0-3	17.85%	6.81%
4-5	64.70%	45.83%

s= sensitivity.

We concluded that telemedicine using smartphone images for trichiasis patient follow-ups is impracticable and will not benefit the population treated at our oculoplastic outpatient clinic due to the low diagnostic sensitivity and interrater agreement of trichiasis identification using this tool.
